# Out of Sync? Rare Genetic Disease and the Chronopolitics of Care

**DOI:** 10.1111/1467-9566.70106

**Published:** 2025-10-14

**Authors:** Catherine Coveney

**Affiliations:** ^1^ Criminology, Sociology and Social Policy Loughborough University Loughborough UK

**Keywords:** care, caregiving, chronopolitics, crip time, disability, lived experience, noonan syndrome, rare genetic disease, temporalities

## Abstract

Drawing on the experiences of parents of children diagnosed with Noonan Syndrome, I examine how living in and between multiple temporalities of care impacts parents’ sense of temporal autonomy and social inclusion. Employing the concept of ‘crip time’, I connect everyday choreographies of care with their temporal politics to analyse *the chronopolitics of care* in the context of rare genetic disease, crafting theoretical synergies between the sociology of health and illness, critical disabilities studies and the sociology of temporality. I argue that care time *is* crip time, requiring parents to juggle competing temporal rhythms that deviate from the chrononormative time order. Parents describe good care as making time and giving time to be with their child to meet their embodied care needs. Meanwhile, the inflexible and unpredictable nature of medical time can be experienced as oppressive and disruptive to the family's care rhythms. Temporal conflicts can create a sense of disconnection, leaving family's feeling out of sync, socially and emotionally. I suggest the need for a new focus on the chronopolitics of care within formal and informal care relations, to support parent carers to regain their temporal autonomy and a regain a shared sense of time and community.

## 1 Introduction

Supporting carers is a global issue and a collective societal responsibility. There are more than 63 million unpaid carers globally (IACO 2018), that is, people who are providing care for an ill, older or disabled family member or friend. Recent census data suggests this equates to over 5.8 million unpaid carers in the UK. Dubbed ‘a silent workforce’ (The Lancet 2024), around 35% are caring for a disabled child (U. K. Carers 2023). Carers overwhelmingly report feeling invisible, unsupported and disconnected (E. Carers 2020).

Care is a long‐standing concern within sociology, recognised as intrinsic to our sociality (Segal 2023). As an embodied, emotional and relational practice, care is at the heart of what makes us human. Rather than being regarded as a burden, the interdependent essence of caring relationships is emphasised (Chatzidakis et al. 2020). Care is something we all provide, and all require (Tronto 1998). Yet, *care work* can, and often is, devalued within a capitalist and patriarchal order (Segal 2023). Recently, there has been a renewed focus on temporalities of care in relation to health and illness. Within this body of work, care practices—the everyday doing of care—are a key focus.

In this paper I use the concept of ‘crip time’ to analyse the temporal regimes of parents caring for children with a rare genetic condition—Noonan syndrome (NS). Connecting everyday choreographies of care with their temporal politics, I analyse what I refer to as *the chronopolitics of care*—the processes and rhythms that underlie the temporal organisation of care practices. In disentangling these temporalities and how they shape the everyday lives of parents, I suggest the need for a new focus on the chronopolitics of care within formal and informal care relationships that centres on both interdependence and autonomy, supports asynchrony and accommodates personal temporalities to enable family carers to regain a shared sense of time and community.

### 1.1 Temporalities of Care

Chronopolitics is the politics of time (Wallis 1970), the processes and biosocial rhythms that underlie the temporal organisation of our daily lives (Mills 2020; Williams et al. 2021). Our lives are structured through time as a vector of power and control (Samuels and Freeman 2021). Within chrononormative modes of temporality, time is conceptualised as linear, managed, controlled and scarce. Speed and acceleration are valued (Virilio 1977). Therefore, time can pass and be experienced differently by diverse groups. Heterotemporality is way of articulating temporality as plural rather than following a unitary trajectory in which are moving towards distinct futures (Hutchings 2007). In analysing how time is socially and politically constructed and experienced, we can critique the chrononormative ways in which our worlds are ordered in space–time. For instance, work on queer temporalities shows us how typical life events can be experienced ‘out of order’ and how temporalities can be indeterminate (Varela and Bayramog 2021). This work provides us with a valuable critique of temporal normativity and the heteronormative imaginings of a life course that follows a linear sequential pattern of progression from birth, through childhood and adolescence, to early adulthood, marriage, reproduction, child rearing, retirement and old age. Emphasis on synchronicity with the dominant temporal order then conceals, erodes, discards and forgets, our individual temporal dynamics, specificities and variances (Amann Alcocer and Delso Gutiérrez 2016).

Time is central to experiences of health, illness and disability. Indeed, illness and disability are defined in and through temporal frameworks in ways that signify departure from normative temporal imaginings of how one should progress through the life course (Harrison et al. 2024). The concept of ‘crip temporalities’ or ‘crip time’ (McRuer 2006) originates from critical disability studies, foregrounding the variable temporalities of disabled embodiment. It can be recognised as *patterns of temporalities*—frequencies, incidences, occurrences, as *speed*, rate and pace—such as slowing down, or needing more time, and as *flexibility* in our expectations of how long things take and when they should happen. Furthermore, Kafer (2013) suggests *time shifts* as people move or think at faster or slower paces than is culturally expected. Bringing together ideas about temporality and futurity, she describes the ‘strange temporalities’ of waiting for diagnosis and being given a prognosis. These liminal temporalities Kafer suggests can ‘cast [one] out of time’ with the present ‘tak[ing] on more urgency as the future shrinks’ (Kafer 2013:56). Crip time, then necessitates ‘a reorientation to time’ (Kafer 2013, 27) that goes beyond hegemonic understandings of normalcy and deviance. In one of her most quoted passages, Kafer argues that ‘rather than bend disabled bodies and minds to meet the clock, crip time bends the clock to meet disabled bodies and minds’ (Kafer 2013: 27). Thus, suggesting that cripping time can engender new forms of solidarities and relationships, compassion and empathy.

Focussing on everyday life with IBS, White (2023) uses the concept of crip time to highlight tensions between the normative temporal rhythms of the social world and lived experience of disability. Her participants detail how their daily routines are shaped by their embodied temporalities—their need to slow down, stretch out or change the time they do things so that they can care for their bodies. She argues that people living with chronic illness can resist and recreate normative temporal rhythms through their relationships and everyday lives, viewing these creative possibilities as acts of resistance that challenge and destabilise normative temporal frameworks.

Although cripping time can enable one to break free of the temporal hegemony, in disabled lives this is often not through choice but out of necessity (Ghosh and Banerjee 2017) or as ‘a mode of survival’ (Price 2021). Reflecting on her own experience of living in crip time, Samuels (2017) articulates how crip time can be a time of grief and mourning for the loss of a heathier self, lost time and the lost years that will not return. It can be broken time, where minds and bodies must bend to new rhythms to move through the world. It is forced breaks, or brakes even, when even the best made plans are put on hold. When all you want to do is move onwards, upwards and forwards, crip time can slow you down and hold you back. As her account illustrates, you are tied to the logics of your body rather than the normative time order of the world around you.

Stevens (2018) reflects on how caring for her disabled partner has changed her own relationship with time. She conceptualises ‘care time’ as a liminal space between crip time and abled time. Carers, she suggests, straddle both worlds, not living fully within crip time, yet inhabiting abled time precariously. Davidson (2020) furthermore, illustrates how caring for a disabled child can transform both how parents experience time in the present and the futures they imagine. Medicine, on the other hand, Kafer argues, is premised on a ‘curative time’ (Kafer 2013; 27), an imaginary that both expects and assumes intervention in attempts to re‐order our bodies within a chrononormative temporal framework. Thus, the lived experience of disability exists in tension with the linear temporal imaginings of illness followed by recovery that dominate biomedicine (Samuels and Freeman 2021).

### 1.2 Temporalities of Healthcare

Healthcare is built on notions of control, regulation and surveillance, constructing good bodies and attempting to discipline deviant bodies within underfunded, strained and compromised systems of care (Cook and Trundle 2020; Ward et al. 2022). Care, in the context of healthcare is an elongated temporal practice, consisting of a plurality of distinct temporal rhythms that may clash, and are difficult to disentangle (Baraitser and Salisbury 2020). For example, in their study on Autism care in Israel, Zarhin and Asher (2024) draw attention to the temporal politics of medicalised healthcare, where the often‐slow pace of conventional medicine sits at odds with parents' sense of urgency to improve care for their child. Likewise, in their study on the temporal politics of dementia care in England, Scotland and Sweden, Ward et al. (2022) detail how access to formal care necessitates surrendering one's temporal autonomy to a ‘chronometric routine’, where waiting and delays are common, care budgets are being slashed and services outsourced to multiple providers. Decisions regarding frequency and duration of formal support are often out of a patient's control, creating a sense of disempowerment. Meanwhile, Cluley et al. (2024)'s research with people living in the UK with end‐stage kidney disease demonstrates how the organisation of medical care around a linear chrononormative conceptualisation of time can be disruptive, restrictive and frustrating for those living with chronic illness. They describe how patients face challenges ‘bending their bodies’ to meet inflexible treatment schedules set by the treatment centre, perceiving these as taking away their freedom and the normality of the lives they once lived.

Baraitser and Salisbury (2020) suggest that ‘giving time’ can be a form of giving care, highlighting the significance of ‘being with’ someone in the present moment. Good care then, not only takes time, but is difficult to measure by the logics of the clock. Rather, the unbounded nature of care practices means that time unfolds at different scales, speeds and tempos (Harrison et al. 2024) across different events, relationships, places and spaces (Cluley et al. 2024). Good rhythms of caregiving should be in sync with the needs of the person receiving care (van Pijkeren et al. 2024), where a sense of inclusion or belonging is linked to feelings of synchronisation (Glavind 2023) not only within the relational dynamics of caregiver and care receiver but also more broadly. Shared social rhythms can create a shared sense of time and community, providing ways for individuals to coordinate their actions (Weller 2023) and thus unifying life experiences locally, or even globally.

Parent carers come to disability in a relational way, their lives intertwined and entangled with those of their disabled children (Davidson 2020). The experiences and realties of parents managing complex care for children with rare genetic conditions is not well researched (Currie and Szabo 2019). However, the research there indicates that constraints on time are a dominant feature of these parents' lives. Parents describe having to juggle their child's care needs with competing temporal demands of family life and other relationships. Temporal conflicts can arise as they navigate between spheres of paid work and caring (Maher 2009). On‐going medical needs punctuate the night, impacting on sleep patterns and leading to constant tiredness, fatigue, exhaustion and stress (K. Coveney and Lambert 2023; C. Coveney and Salem 2025). Parent carers describe having ‘no time’ for themselves, not enough time for others, too much to do and having not enough time to do it in (Mcmullan et al. 2022; Page et al. 2020).

Drawing on an empirical study with parents of children diagnosed with Noonan syndrome (NS), in the remainder of the paper I analyse the embodied and everyday temporalities of caregiving, arguing that *care time is crip time*. Through the lens of ‘medical time’ I examine how participant's experiences of caregiving are shaped by the temporal politics of medical care. I discuss how living in and against the chrononormative time order can lead to temporal dislocations where desynchronisation with dominant social rhythms can make it difficult to sustain employment, maintain routines or participate in social life.

## 2 Methods

Data were collected via a mixed methods study with 67 families living with Noonan syndrome. NS is one of over 7000 ‘rare diseases’ that collectively affect over 300 million people worldwide. With an estimated prevalence of around 1:2000, is it one of the most frequently occurring ‘rare’ genetic conditions. It is also one of the most complex rare syndromes in terms of both its genetic and phenotypic profile (Genetic Alliance 2024). Typically presenting in childhood, the multi‐system syndrome is linked to at least 14 gene variants (Murray 2023) and is one of the leading syndromic causes of congenital heart disease (Sun et al. 2022). NS is commonly associated with an array of comorbidities ranging from learning difficulties to renal and lymphatic anomalies, bleeding disorders, childhood cancer, short stature, Autism and ADHD (Naylor et al. 2023; Roberts 2022; K. Coveney and Lambert 2023), although clinical presentation is variable. Thus, NS provides a powerful exemplar of lived experience of caring for a child with a rare genetic condition, while offering valuable insights into the wider realities of informal or family caregiving.

The Living with Noonan Syndrome project received ethical approval by Loughborough University ethics committee in April 2022. An anonymous online survey was distributed via social media to two NS‐specific Facebook groups and by email to Noonan Syndrome Association UK (NSA) members, between June and November 2022. The online survey was designed to collect data on the demographics and specific support needs of families and to recruit participants for a follow‐up qualitative interview. Data excerpts presented in the article are drawn from the interviews. Analyses of survey data is presented in more depth elsewhere (K. Coveney and Lambert 2023; K. Coveney and Salem 2025).

Approximately 10% of NSA members completed the survey. Thirty‐five participants expressed an interest and were invited to participate in an interview. However, recruitment was challenging. Seventeen participants withdrew from the study at this stage for health reasons or scheduling difficulties. Thirteen mothers and five fathers of children with NS were interviewed between September 2022 and October 2023. Interview participants were aged between 29 and 76, with an average age of 48. All identified as White British. Ten participants had children 0–10 years of age. Four participants were caring for a young adult with NS, aged 16–22. One participant reflected on caring for her son who died aged 18 and one was a full‐time carer of a dependent adult child (aged 25–30). The remaining two participants were caring for their adult child (aged 30+) and their teenage grandchild. Four participants were in full time employment, seven worked part‐time and one was retired. The remaining participants identified as full‐time carers. Despite efforts to recruit a diverse sample, these data do not represent a universal experience of living with NS. For example, adults with NS who live independently and manage their own healthcare were not interviewed. Nevertheless, these data provide us with a rich and detailed insight into the lived experiences of parents caring for children with a rare genetic condition, a demographic that has yet to receive significant scholarly focus.

Participants chose who was present during the interview and whether they were interviewed individually or as a couple or family group. Three interviews were carried out in mother/father pairs. The couple dynamic shaped how candidly participants spoke about their relationships and the gendered division of care within their family. In several instances, another family member (e.g., child's grandparent) was present in a supportive role and did not participate in the interview. During three interviews, children were present for a time. Although children spoke to their parents and were introduced to the researcher, these exchanges were not included as research data. The presence of children in the interview space impacted on the tone and topic of conversation which tended to shift towards discussion of positive aspects of family life with NS rather than its challenges.

Photo‐elicitation was used to guide interview discussions. Each participant submitted up to three photographs in advance of the interview as visual representations of what living with NS meant to them. They were asked follow‐up questions, in no order, relating to the content of their photographs and around the themes of diagnosis, caregiving, relationships, reproductive decision‐making and social and emotional support. The photographs provided a mechanism for participants to emotionally prepare for the interview (Fearon 2019), as well as giving them some control over its content (Aldridge 2007). An implication of invoking this method was the disruption to hegemonic temporality in the participants’ accounts. Instead of producing a narrative account beginning with a pregnancy or birth story and covering milestones to the present day, each participant's story began and ended at a different time and place, unfolding around events they deemed to be of most significance to them. Consequently, in participants accounts there was often no defined beginning, middle and end. Narratives entered temporal loops and were temporally ambiguous as participants readily switched between reflecting on the past, describing the present and contemplating the future.

Interviews were recorded, transcribed and then coded in NVivo 14. Using reflexive thematic analysis, I took an interpretative sociological approach to generate themes (Braun and Clarke 2019). This involved development of a theoretically informed coding frame with a focus on temporalities and care. Through an iterative process of reading and re‐reading transcripts, I identified language used to demarcate time; patterns of temporalities, such as frequency, incidence, speed and pace; orientations to time including past, present and future and events‐based temporalities, such as remission, relapse and repetition. Data extracts were grouped together based on their main topics, and congruent codes were connected to generate and develop themes. In the following Section 1 discuss empirical themes relating to (i) care time as crip time, which focuses on embodied temporalities, orientations to time and everyday care practices and (ii) medical temporalities, which outlines three overlapping temporal regimes of medical care—hospital time, waiting time and crisis time.

## 3 Findings

### 3.1 Care Time as Crip Time

In this section, I centre on parents' experiences of caring for their disabled child in time through the lens of crip time. Data show how embodied temporalities shape orientations to time, how care unfolds at varied tempos, and how clashes with chrononormative structures of school, work and society create temporal dislocations.

#### 3.1.1 Embodied Temporalities and Orientations to Time

In relation to embodied experiences of disability in time (Kafer 2013), parents are caring for a disabled child who does not follow a normative life course trajectory (Samuels and Freeman 2021). Instead of milestones that mark hegemonic life course transitions, life happens at different tempos. As Amy, mother to a 10‐year‐old child with NS describes, timeframes are often extended and expanded. There is a sense that the child will ‘get there’ in their own time:

The milestones that you take for granted as a parent took a really long time. Standing [and] walking took forever because he has low muscle tone. Riding a scooter, riding a bike, learning to swim, reading took him until he was eight, but who cares? In the whole scheme of things, he met those milestones.

Through these data, we get a sense of how time can stand still (Davidson 2020). Families become desynchronised from the dominant temporal order as children remain dependent on their parents for periods that extend beyond chrononormative imaginings of progression through the life course (Harrison et al. 2024), thus necessitating new temporal imaginings of the future. This is captured by Michelle as she reflects on the support her daughter, now a young adult, requires with personal and social care:

You see people who have children the same age and they're all off doing independent things … She's 22. You're still stuck saying, have you cleaned your teeth? All this kind of stuff. And you think this is just going to go on forever.

For others like Joan, whose adult daughter and grandchild both have NS, care needs may change over time, yet requirements for parental support persist. Good parenting then, is not premised on preparing children for independence. Instead, ongoing mutual dependence is accepted and normalised:

She needs help with organising her money, her paperwork, [with medical appointments] … we are very much part of her life even now and will be all of her life in the organisation of her life and how she's dealing with it.

Others were faced with ‘strange temporalities’ (Kafer 2013, 56) of uncertain prognosis, where declining health necessitated a reorientation towards the present. We can see this in Laura's account as she reflects on her experiences of caring for her son, who died from complex health issues when he was 18 years old:

You really live in the present. You couldn't make plans for six months' time, but you could enjoy today so you'd eat at great pleasure and [enjoy] the smaller things in life like going for a walk … there's a value in appreciating what you've got and not yearning after other things in the future.

Likewise, in the extract below Helen talks about how she felt after her now 2‐year‐old son was diagnosed with NS:

You're thinking my child is probably going to be lifelong dependent with me. He's going to get every single [medical complication]. He might not survive … But when I look at the minute, I know he is doing well. Without comparing him to anybody else, he is doing well at the minute.

For some participants then, adopting a temporal perspective oriented towards the present offered a way to cope with the uncertainty of the future.

#### 3.1.2 Temporal Organisation of Everyday Care

Viewing the temporal organisation of everyday care through the lens of crip time reveals how processes and rhythms that underlie everyday care practices deviate from the chrononormative logics of clock time. In parents accounts, good care meant slowing down together to centre meaningful care, connection and validation of the child's care needs. Everyday acts of care—feeding, washing, travelling from one place to another, falling to sleep—can all take longer. Moreover, there is more care to give and more care to fit into the day. These ‘extra’ everyday caregiving tasks included preparing special diets, assisting with personal care, administering various medicines and other therapies at home, such as speech and language therapy, physiotherapy and occupational therapy exercises.

For parents caregiving is characterised by temporalities of repeated sequences, patterns and routines, shaped by their child's ongoing care needs that span the days and nights. Feeding difficulties, atypical sleep patterns and managing pain were aspects of everyday care that featured heavily in parents’ accounts. Good care often necessitated wakeful nights, where parents had to remain vigilant and were responsible for ensuring their child's wellbeing and safety. For example, participants whose children were tube fed talked about the extra levels of care required when feeding their child through the night, such as checking the nasogastric tube had not become dislodged, managing a feeding pump and responding to episodes of vomiting:

He was on an overnight feed and then in the daytime we’d try to encourage him to eat normal food. The nights were horrendous. He was vomiting a lot—it was a nasogastric tube—so having to reinsert that because it often came out.

(Laura)

Families spoke about needing to slow down, stretch out and change the time they did things to operate at their child's pace. Richard, for example, explained how he gives his 6‐year‐old daughter several medications every morning via her gastrostomy tube. Every evening, he must give her several more medications before she goes to bed. The extra time she needs for medications or to eat causes time pressure; the family feels like they are always rushed or running late as they fall out of sync with clocked time:

We are quite often late for school; every morning is a rush. It takes longer for her to eat; she needs more time. If she eats too fast, she vomits and then she loses valuable calories that she needs to grow. She has [three medications] we try and fit it in the morning, it's not always quick. We do [four medications]at bedtime, it’s a lot of pressure because she needs to do all of the school activities that normal children have to do.

In conceptualising care time as crip time we can see how it can be embraced by family carers, as they accept that good care means that care takes as long as it takes. Their non‐normative temporalities become enfolded into the patterns of their daily lives (Mattingly 2014), as they build their own routines around their child's embodied care needs and find their own rhythms. Therefore, as Richard's account above highlights, temporal conflicts arise as families attempt to fit in extra care needs alongside the chrononormative rhythms of everyday life. Indeed, most parents experienced some degree of time poverty, leaving them feeling exhausted, fatigued, constantly tired, burnt out and left with little time for self‐care. Richard goes on to say:

We're alone quite often. There are barriers for relationships, you lose a lot of friends. It's difficult to make new ones. You don't have the time; we can't always go to everything to meet other parents because we have other challenges. You don't really get that [emotional support].

His account indicates how when families crip care time and live by their own temporal rhythms shaped by their child’s embodied care needs, it can be difficult to synchronize with wider shared social rhythms. Richard's rhythms of care were not recognized by others—school, employers, friends—whose temporal expectations for what his family should or could do and when did not shift. Many participants expressed feeling like they could not keep up or did not fit in, leaving them feeling isolated, alone and emotionally unsupported.

As evidenced in Meg's account below, amidst the daily routines of medical care, participants can find solace in pockets of stability and ‘normal family life’. However, crip time can also be viewed as ‘a mode of survival’ (Samuels 2017), a fragile stability that exists within the strange temporalities (Kafer 2013) of living with a rare genetic syndrome where the future is uncertain.

You're living on a knife edge a lot of the time, you're grateful when she has good days and good spells. But you always know that there is inevitably something around the corner. You can never be complacent, never fully relax; you're waiting for the next thing.

(Mother to a 6‐year‐old child with NS)

In the data extract below Edward and Joan explain how their daughter, now in her late thirties, has undergone multiple cardiac surgeries. Even when early surgeries are successful, complex cardiac defects are not cured. The prognostic trajectory (Timmermans and Stivers 2018) is an uncertain period of relative medical stability that coexists alongside anticipation of deterioration and the need for future surgeries. Thus, time is experienced as patterns of remission, relapse and repetition. There is no end to care time as their child's care needs are enduring and ongoing.

Joan She was nine when she had her first open heart surgery

Edward They took the valve out, but they didn't replace it. She had a second open heart surgery [in her early twenties]. They put a human valve in, and we were told probably 15 years max for that to last

These parents, then, can never make the switch to ‘abled time’ and leave crip time. For them, care time means living with constant uncertainty and unpredictability for the future, while focussing on providing good care in the present.

### 3.2 Medical Temporalities

When living with a complex syndrome such as NS, medical care involves simultaneous attentiveness to multiple comorbidities. In addition to managing medical needs at home, its medical complexity entails attending multiple specialist clinics (typically spread across different geographical sites) for treatment and lifelong medical monitoring. Repeated and prolonged hospitalisations for planned surgical interventions and unplanned emergency admissions featured heavily across the dataset. Thus, the hospital looms large in daily life, not only as a place of care but also a spectre, reflecting its pervasive presence in family life no matter their child's age. As discussed earlier, when it comes to medical care, a plurality of temporalities is entangled. This section examines three medical temporalities—hospital time, waiting time and crisis time—to illustrate how the temporal incommensurability between cripped care time and the chrononormative structures of medical time can be a source of temporal dislocation for families living with NS.

#### 3.2.1 Hospital Time

Hospital time describes the institutional organisation of time; its rhythms anchored in the chrononormative, linear frames of clock time and calendar time. Dates and times for hospital appointments are set by scheduling systems that prioritise institutional efficiency and staff availability, creating a repetitive and predictable rhythm for staff. For families, however, lived experience of hospital time can feel unpredictable. Patients are given little advance notice of dates and times for appointments and planned admissions. They have limited temporal autonomy nor agency to change their allocations. Like Sarah, who has a 9‐ year‐old child with NS, all participants described spatio‐temporal challenges of managing multiple medical issues within a fragmented and uncoordinated healthcare system:

He's got 15 consultants at three different hospitals…his main hospital is in [City 1] which is over an hour's drive away … Last week we went to see a spinal doctor in [City 2] and then he went to see his stoma nurses in [City 1]. And you have to tell them both exactly the same story because there's no communication and no one knows what's going on with him.

Hospital time can clash with families' daily routines and rhythms of care, such as school drop‐offs, and working hours that have been forged around their child's embodied care needs. In order to prioritise medical care and ensure their child gets the care they need, parents described having to bend to hospital time:

Clinics are only held on certain days of the week. Your employer may go ‘can you do a different day?’ You can't do a different day. That's when the cardiologist or whoever has their clinic. You have to make that appointment. Otherwise, you feel as if you're letting [child] down.

(Richard, father to a 6 ‐year ‐old child with NS)

Thus, the incongruity of temporal logics can be a source of temporal dislocation, with work time and care time being subordinated to hospital time. As Stacey reflects, the unpredictability of hospital time poses significant challenges for parents to maintain paid employment. Siblings' routines are also upended as the family's rhythms of care are shaped by medical appointments, where inflexible appointment schedules can take away a family's sense of normality.

You can’t hold a job when you’ve got to keep going to the hospital… We had a lot of hospital appointments, and my eldest child had to come with us a lot of the time. I felt like they weren't having a normal life.

(Mother to a 16‐year‐old daughter with NS)

Planned hospital admissions for surgery, investigative procedures and diagnostic tests were a regular occurrence for all participants, although the frequency differed between families. Like Helen, who has a 2‐year‐old son with NS, all the parents interviewed described spending endless days and nights sitting by their child's hospital bed, sleeping in the hospital, being with their child:

Anytime he’s in hospital, I’m there. His open‐heart surgery, I spent the night in intensive care by his bed. Well, all of that week.

In some instances, hospital admissions lasted a day or two, whereas in others, this could mean parents spending weeks, or even months living in the hospital. Laura was one of several participants who described experiencing extended hospital in‐patient stays with their child. Her account emphasises how interdependence sits at the heart of caring relationships, with parent and child viewed as team, creating a shared rhythm of care:

I was with him all the time … the longest was in 2020, when he was in for eight weeks with endocarditis. They have a bed for the parent, so I just stayed there with him. We were kind of a team.

The data also show how hospital time routinely displaces social time. Participants described rescheduling holidays, planned outings and missing school plays, birthdays, Christmas celebrations and other social events to attend hospital appointments. Although appointments can be rearranged, when faced with overbooked clinics, long waiting lists and ‘slow care’ (Zarhin and Asher 2024) a sense of urgency underlies every appointment. Parents often felt that they had no other choice but to submit to these temporal regimes. This is exemplified in Meg's account below:

[6 yr old daughter] had to go for a scan on her birthday…I just thought that is kind of a metaphor for her life—for our life—because there’s no day off […] trying to rearrange any appointment within the NHS is not an easy thing. Fundamentally, she needed that scan […] you can't ever really escape the hospital.

Some families stopped planning social activities altogether, anticipating their displacement by hospital time. This is discussed further in the next section.

#### 3.2.2 Waiting Time

Waiting time is a pervasive, if not defining, aspect of the chronopolitics of medical care and a product of how the NHS distributes, allocates and controls time. Participants described waiting for appointments, test results, diagnoses, referrals, and treatment decisions. Waiting is another strange temporality (Kafer 2013), a liminal space characterised by temporal inequality; while hospital time ticks to the institutional clock, care time must pause, stretch and bend to absorb the delays of the system. Waiting time can be experienced as broken time, where time moves at a slower place. As Sam's account demonstrates, this can be frustrating for families who are left waiting for vital information:

It has been frustrating … the time it takes for appointments to come through, the time it takes to then get a medical letter … we had a recent MRI and we were basically waiting, literally shitting ourselves. Has her tumour grown? And they did never [tell us].

(Father to a 7‐year‐old child with NS)

When living with NS there is no end to waiting as the future is uncertain. Families were waiting for the next operation, the next crisis, for healing, recovery or capacity, or for deterioration, regression or decline. In the extract below Laura reflects on her son's medical care as a ‘lifelong prospect of waiting’:

He was always waiting for another operation. That sense of waiting and waiting and waiting was very hard…it was never, ‘you'll have this operation then you can get on with your life’. It was ‘you have this operation and hopefully your heart will function a bit better for a few more years. And then I'll probably have to do more surgery’. It was like a life lifelong prospect of waiting.

She went on to explain how living with an unpredictable prognosis and the uncertainty of when the next hospital appointment or admission would be shaped her family's rhythms of care. For Laura, this meant a reorientation to time, where she felt unable to make plans for the future:

You can’t make plans, you can’t say, ‘in six months’ time I will go on holiday’ because you never knew when you would get a date for the operation.

Waiting time often clashes with parents perceived sense of urgency and can create a sense of powerlessness, where parents feel like their child has ‘fallen through the cracks’ of the system and been forgotten. Several participants, however, refused to submit to the slow temporalities of medical care. Like Sarah, they engaged in acts of temporal resistance, chasing appointments, negotiating waiting lists, fighting for access to treatments and trying to speed up medical time:

I’m constantly chasing for appointments, he falls off the radar, like ENT for his ears, every single year they forget about him. I have to ring up and ask for the appointment myself…Growth hormone treatment hasn't even been offered to us yet…you have to fight for it.

Other participants shared similar stories of their attempts to circumvent long waiting lists to see allied health professionals such as physiotherapists, speech and language or occupational therapists.

#### 3.2.3 Crisis Time

Crisis time, another pervasive temporality of medical care, is an event‐based temporality characterised by accelerated bursts of urgency that punctuates families' everyday rhythms of care. Crises for my participants included emergency surgeries, health deteriorations, new or unexpected complications that appeared without warning, and acute hospitalisations. In extract below, Noah and Louise explain how common viruses and infections can impact their now 6‐year‐old son in severe and unexpected ways, resulting in five emergency hospital admissions during the first year of his life alone:

Louise That first year was a succession of dashes to hospital… [It was the] first time ever in my life [I've had to] dial 999. We had the blue lights and the ambulance to come and get [child] because he was struggling with breathing and concern around oxygen levels

Noah The five times were just the emergencies, not the ‘every three‐week’ visits into hospital for that first year of his life

Crisis time is marked by an acceleration in medical care. Medical time speeds up as decisions on intervention, treatment and care cannot wait. We can see this in Meg's account below, as she describes her daughter being diagnosed with intestinal malrotation, aged 4:

… they [saw] the state of her intestines, then it's like, ‘Oh my God, panic got to get this kid in [to hospital]. It's an emergency!’ And just the realization that she could have died at any point … We were in hospital for a month, her body wouldn't go back to normal for a really long time.

The immediacy of the crisis overshadows all else as families must live in the present and focus on getting through *this* surgery, *this* infection or *this* hospital admission. As evident in Amy's account, they often found themselves spatio‐temporally dislocated and socially disconnected, living in limbo in the hospital away from their support networks:

[Child] got called in to have an emergency stoma fitted. I had a five‐week‐old baby that I had to leave with my mum while me and [child] went and stayed in [hospital in a different city] for six days.

Emotionally, there is also a sense of time standing still. As Richard reflects, although the world moves on around you, during crisis time, time is frozen in the present moment:

All of the operations, the fear, the anxiety … sat in the room waiting for [the doctor] to come out … everything slows down and then you find out that she’s ok, or that operation didn't go well, that she very nearly didn’t make it through the surgery. At the time you try to do whatever you can to get through that situation.

Collectively, participant's accounts gave a sense of how these families are living through repeated medical crises—life and death situations that are not isolated incidences, but reoccur at frequent, yet less predictable intervals. For example, Michelle described the ongoing impacts of epilepsy on her daughter's life. She experiences over 25 seizure episodes each year and requires emergency hospital admissions every couple of months, despite being on medication:

They keep her in [hospital] to ensure that she is recovering. [Last month she] was in hospital for her birthday … When she’s had all those seizures and all that medication, it takes her several days to be back to herself. We cancelled all the plans we'd made for her birthday.

The urgent speed of crisis time contrasts sharply with the slower pace of routine hospital time. As we can see in Michelle's account above, time stands still as plans are cancelled, work ceases, daily routines are suspended. In the extract below Louise, mother to a six‐year‐old child with NS, describes leaving her job, finding work time incommensurable with hospital time and crisis time:

I gave up work for the first two and a half years of his life … it just wouldn't have worked with having to go to the hospital for emergency situations, he has an awful lot of checkups for his issues as well.

Furthermore, crisis time also operates as an anticipatory temporality (Timmermans and Stivers 2018). The anticipation of future unexpected and frequent medical crisis shapes everyday care practices, as families prepare for future crises. In the extract below, Dave describes the careful consideration that now goes into every family decision such choosing holiday destinations and locations that are near to a hospital, in case of unforeseen emergencies:

[NS] affects her life and obviously affects our lives. If we go on holiday, we always check where's the nearest hospital. We used to like going to remote places—we don't do that anymore. We are cautious about going abroad as you want to make sure that if you end up in a hospital you know you can speak the language.

(Father to a 22‐year‐old child with NS)

Thus, carers are not only temporally bound, but also spatially, to maintain connections to life saving medical care.

## 4 Discussion

Employing the concept of ‘crip time’, I have connected everyday care practices with their temporal politics to analyse what I have referred to as the chronopolitics of care in the context of rare genetic disease, crafting theoretical synergies between critical disabilities studies, the sociology of health and illness and the sociology of temporality. Crip time foregrounds the variable temporalities of disabled embodiment and attunes us to heterotemporalities that depart from the chrononormative imaginings through which our worlds are ordered. Crip time is living out of sync with society's rhythms and routines. It is characterised by multiple entangled temporalities (Chazan 2023), shifts in tempo that cannot be reduced to slowness and extended time (Kafer 2021), or the need for ‘more time’. Rather, crip time is the disruption of normative temporality, it challenges both how we make sense of time (Bierdz 2023) and how we experience it through our care practices. Drawing on McRuer's (2006) formative works on ‘crip theory’, Gauthie‐Mamaril (2024) reminds us that engaging in crip practices or perspectives is not contingent on identifying as disabled. I argue that for these parents at least, care time is crip time; that caring for disabled children necessitates the creation of non‐normative rhythms of care as ‘a mode of survival’ (Price 2021).

Parents’ accounts of providing everyday care for their children emphasised departure from the temporal hegemony in several ways. Parents are caring for a disabled child who does not follow a normative life course trajectory (Samuels and Freeman 2021). Instead of milestones that mark chrononormative life course transitions, life is characterised by stops and starts, intervals and, for some, abrupt endings (Samuels 2017). Cripping care time is wakeful nights and sleepful days. Timeframes are expanded as good care takes as long as it takes. Extra time is required as everyday acts of care take longer and there is more care to fit into the day. Making time and giving time to be with one's child are fundamental aspects of everyday care practices, where good care is love, empathy, compassion and connection; it is emotional, relational, embodied, interdependent (Segal 2023). As these data show, care time is also desynchronisation and disconnection. It is stopping and time out, watching life pass by (Price 2021). Care time can be exhausting, expensive, frustrating, emotionally distressing and stressful. As Samuels and Freedman (Samuels and Freeman 2021, 247) remind us ‘crip time is not easy, it is not fair, it cannot be reasoned with’.

The analysis presented emphasises how caring for a disabled child does not conform to linear models of care and recovery; rather, it is characterised by rhythms of recurrence and repetition (Kafer 2021), a reality that requires wider social recognition. Within the variable temporalities of disabled embodiment, care needs are cyclical, repetitive, enduring and ongoing. The data show how these non‐normative temporalities of care become normalised within the family as they find their own rhythms (K. Coveney and Lambert 2023; Mattingly 2014), which come to characterise periods of stability that families cherish. However, this fragile stability exists in tandem with the uncertainty and unpredictability of when it might be punctuated by the next crisis. With a lifelong genetic condition like NS, there is no end. Thus, marking a stark departure from the linear temporal imaginings of ‘illness, treatment and recovery’ that dominate biomedical curative imaginaries (Kafer 2013).

Taking a chronopolitical lens, meanwhile, provides the tools to account for both the varied and nonhegemonic temporalities of disabled lives, and how these temporalities are socially and politically produced. The analysis indicates how the temporal rhythms of care that families forge clash with and are fractured by institutional times, where normative chronopolitics are enforced. These data illustrate how the cripped rhythms of caregiving families create are often temporally incommensurable with the rigid and inflexible nature of hospital time, work time, school time and social time, which are predominantly structured through linear chrononormative modes of temporality.

In relation to healthcare, normative chronopolitical regimes of clock time and calendar time structure the scheduling of hospital appointments, clinics and routine surgeries. This is shaped by the temporal politics of an overstretched, underfunded and fragmented healthcare system, where unpredictability and a slow pace of care sit in tension with parents’ perceived urgent need (Zarhin and Asher 2024). Thus, waiting time (Baraitser and Salisbury 2020) dominates experiences of routine medical care. Long‐term waiting can be painful as individuals must accept and cope with its uncertainty and liminality (Baraitser and Salisbury 2020). Crisis time—accelerated bursts of urgency—meanwhile repeatedly punctuates and disrupts families established rhythms of care time resulting in temporal dislocations from social time and work time.

Parents described having little temporal autonomy over the timing of the medical care their child received. Some described engaging in acts of temporal resistance in their attempts to speed up waiting times, fighting and pushing for medical attention in a climate of medical scarcity. Often though, they felt they had no choice but to bend their informal rhythms of care to ensure their child received the medical care they required. Thus, these data demonstrate how difficult it is for families living outside of the chrononormative temporal order to ‘bend the clock to meet [their] disabled bodies and minds’ (Kafer 2021).

Although cripping care time engenders creative possibilities for new ways of being in the world (Davidson 2020), it simultaneously requires desynchronisation from the hegemonic time order. Cripped rhythms of care, then, can also sit in tension with wider collective social rhythms that create a shared sense of belonging and community (Glavind 2023). Disconnection from the shared rhythms of social life can leave families feeling ‘out of sync’ with the world around them, invoking a sense of isolation (Lassen et al. 2020) and exclusion (Yarskaya‐Smirnova and Yarskaya‐Smirnova 2021).

Care is a collective responsibility and supporting unpaid and family carers is a significant and growing global issue. Focussing on the *chronopolitics of care* within formal and informal care relations can shift our attention to the wider structural and political changes, infrastructures and resources that are necessary to lay the ground for more caring societies.

Socially, inclusive spaces, that accommodate personal temporalities, are vital for people to regain a shared sense of time and community. Within systems of formal care and employment, there remains a pressing need for practitioners and employers to bend their clocks (Kafer 2013) to meet disabled individuals and their family's needs. Supporting asynchrony and providing flexibility in expectations over how long things take, or when they are done can give parents caring for a disabled child a greater sense of temporal autonomy. Furthermore, for systems to change there needs to be a deeper appreciation of what the realities of informal care for a disabled child entail. This includes greater acknowledgement that caring for a disabled child does not conform to linear models of care and recovery, that interdependence sits at the heart of care and caring and that making time and giving time to be with one's child are fundamental aspects of everyday care practices. Dismantling the chronopolitics of medical time is no easy feat. However, increased recognition of how the chrononormative temporality of hospital time is experienced, and the tensions that its rigid and inflexible structure creates with rhythms of informal care practices will help to foster a healthcare system that is both more care‐full and responsive.

## Author Contributions

**Catherine Coveney:** conceptualization (lead), data curation (lead), formal analysis (lead), funding acquisition (lead), investigation (lead), methodology (lead), project administration (lead), supervision (lead), writing – original draft (lead), writing – review and editing (lead).

## Conflicts of Interest

The author declares no conflicts of interest.

## Data Availability

The data that support the findings of this study are available on request from the corresponding author. The data are not publicly available due to privacy or ethical restrictions.
